# Phytosterols Supplementation Reduces Endothelin-1 Plasma Concentration in Moderately Hypercholesterolemic Individuals Independently of Their Cholesterol-Lowering Properties

**DOI:** 10.3390/nu12051507

**Published:** 2020-05-22

**Authors:** Angela Oliveira Godoy Ilha, Valeria Sutti Nunes, Milessa Silva Afonso, Edna Regina Nakandakare, Guilherme da Silva Ferreira, Renata de Paula Assis Bombo, Ricardo Rodrigues Giorgi, Roberta Marcondes Machado, Eder Carlos Rocha Quintão, Ana Maria Lottenberg

**Affiliations:** 1 Laboratorio de Lipides (LIM10), Hospital das Clinicas HCFMUSP, Faculdade de Medicina, Universidade de Sao Paulo, Sao Paulo 01246-903, Brazil; angelailha@hotmail.com (A.O.G.I.); valeriasutti@gmail.com (V.S.N.); milessafonso@gmail.com (M.S.A.); enakonda@usp.br (E.R.N.); ferreira.gui@hotmail.com (G.d.S.F.); rebombo@terra.com.br (R.d.P.A.B.); rmarcondesmachado@yahoo.com.br (R.M.M.); equintao@terra.com.br (E.C.R.Q.); 2 Laboratory of Cellular and Molecular Endocrinology (LIM-25), Faculdade de Medicina, Universidade de Sao Paulo, Sao Paulo 01246-903, Brazil; rrgiorgi2@hotmail.com; 3 Faculdade Israelita de Ciências da Saúde Albert Einstein (FICSAE), São Paulo 05521-200, Brazil

**Keywords:** phytosterols, endothelin-1, cholesterol, inflammation, diet

## Abstract

Experimental and clinical studies have demonstrated the effect of phytosterols (PS) on reducing plasma levels of cholesterol and LDL-c, but the effects of plant sterols beyond cholesterol-lowering are still questionable. Since inflammation and endothelial dysfunction are involved in the pathogenesis of atherosclerosis, this study aims to evaluate the effect of PS on biomarkers involved in atherosclerosis progression and whether these effects are independent of alterations in plasma LDL-c levels. Thirty-eight moderately hypercholesterolemic volunteers (58 ± 12 years; LDL-c ≥ 130 mg/dL) were randomly assigned to consume 400 mL/day of soy milk or soy milk + PS (1.6 g/day) for 4 weeks in a double-blind, placebo-controlled, cross-over study. Blood samples were collected and lipid profiles and biomarkers for inflammation and endothelial dysfunction determined. The results showed that PS treatment reduced endothelin-1 plasma concentration by 11% (*p* = 0.02) independently of variations in plasma levels of LDL-c. No alterations were observed regarding fibrinogen, IL-6, hs-CRP, SAA, TNFα, or VCAM-1 between placebo and PS-treated groups. Furthermore, PS reduced total plasma cholesterol concentration (−5,5%, *p* < 0.001), LDL-c (−6.4%, *p* < 0.05), triglycerides (−8.3%, *p* < 0.05), and apo B (−5.3%, *p* < 0.05), without changing HDL-c concentration (*p* > 0.05). Therefore, PS supplementation effectively lowers endothelin-1 independently of the reductions in plasma levels of LDL-c, contributing to the comprehension of the effect of plant sterols on endothelial function and prevention of cardiovascular diseases.

## 1. Introduction

Hypercholesterolemia is one of the main causes of atherosclerosis and elicits a cascade of cellular and molecular events leading to endothelial dysfunction, inflammation, plaque instability and cardiovascular events [1,2]. Endothelial dysfunction contributes to the early appearance and manifestation of atherosclerosis in hypercholesterolemic patients [3] and is characterized by increased expression of inflammatory cytokines and adhesion molecules such as intracellular adhesion molecule (ICAM-1), vascular cell adhesion molecule (VCAM-1), and E-selectin [1,4].

Endothelin-1 (ET-1) has a relevant impact on the development of endothelial dysfunction [5], as it elicits vasoconstriction and several deleterious effects including chemotactic and mitogenic properties [6], which culminates in smooth muscle cells proliferation [7]. Moreover, plasma [8] and tissue [9,10] ET-1 concentrations are strongly related to high plasma levels of LDL-c, especially oxidized LDL [11]. Therefore, dyslipidemic patients submitted to nutritional and pharmacological treatments to reduce plasma LDL concentrations, present a reduction in inflammatory and coagulation biomarkers, which contributes to improvement in endothelial function and reduction in cardiovascular events [12,13].

It is well known that phytosterols (PS) reduce plasma total cholesterol and LDL-c concentrations due to molecular actions in enterocytes and hepatocytes and displacement of cholesterol from micelles in the intestinal lumen [14,15,16,17,18,19,20], which increases fecal cholesterol excretion [21]. Moreover, phytosterols induce LDL receptor expression [22], and reduce the susceptibility of LDL to oxidation [16] and, therefore, contribute to the prevention of atherosclerosis development [21,23]. Because of the beneficial effects on the lipoprotein profile, in 2001 the National Cholesterol Education Program Expert Panel (NCEP ATP-III) included phytosterols in the dietary treatment for moderate hypercholesterolemia [24]. However, after the publication of this guideline, some reports have shown that high PS plasma or tissue concentrations could be related to an increase in cardiovascular risk [25,26]. Nevertheless, our previous study showed that LDLr-KO mice fed a high saturated fat diet supplemented with 2 g of PS did not accumulate sterols in the arterial wall and promoted remarkable prevention on atherosclerotic lesion development [23].

Therefore, we evaluated whether PS-feeding can reduce cardiovascular risk in humans by mechanisms beyond the reduction in cholesterol plasma concentration.

## 2. Materials and Methods

### 2.1. Subject Recruitment

Volunteers (*n* = 38; 31 female and 7 male) aged 38–77 years were recruited in the Dyslipidemia Outpatient Unit of the Endocrinology and Metabolism Service from the University of São Paulo, Brazil. Staff members of the University of São Paulo were also enrolled in this study. The Clinical Trial was approved by the Ethics in Research Committee of the University of São Paulo Medical School (CAPPesq no. 112/06) and written consent was obtained from each patient. The participants were invited to the screening of body weight and height, and blood samples were collected. The inclusion criteria were: body mass index between 20 and 30 kg/m^2^; TC between 200–300 mg/dL, LDL-c concentrations ≥ 130 mg/dL, and triglycerides ≤ 250 mg/dL. This was a parallel group, double-blind, placebo-controlled, single-center dietary intervention trial. Participants were assigned with a unique number and a simple random sampling was performed by a statistician to allocate the subjects to the intervention or placebo groups.

### 2.2. Blood Sampling

After fasting for 12 h, blood samples were collected in tubes containing EDTA (10 μL EDTA/mL). Plasma was immediately separated by centrifugation (1300 *g*, 15 min, 4 °C; RT6000B; Sorvall Instruments, DuPont Co, Newton, CT, USA), and the following preservatives were added: 0.25% chloramphenicol and 0.5% gentamycin (20 μL/mL), 2 mmol benzamidine/L (5 μL/mL), 10 mmol phenyl-methyl-sulfonyl fluoride/L (0.5 μL/mL), and aprotinin (0.5 μL/mL). The serum was collected for the measurement of fibrinogen and, plasma for HDL measurements. Aliquots of total plasma and serum were stored at −70 °C for further analysis. All measurements were performed in duplicate at the end of each period of the study. All samples from one subject were analyzed within the same analytical run.

### 2.3. Lipids Profile

Plasma lipid concentrations (total cholesterol, HDL-c, and triglycerides) were measured in COBAS MIRA (Roche Diagnostics, Basle/Basel, Switzerland), using commercial kits from Roche Diagnostics (Mannheim, Germany). HDL-c was measured after the apolipoprotein (apo) B precipitation [27] by dextran sulfate and magnesium chloride (1:1) solution (50 µL/500 µL of plasma). LDL-c was calculated according to Friedewald formula [28] and apo-B was measured by a turbidimetric method (Randox Laboratories, Crumlin, United Kingdon).

### 2.4. Inflammatory and Endothelial Dysfunction Biomarkers

Fibrinogen was analyzed according to the modified Clauss method [29], and hs-CRP and serum amyloid A (SAA) were measured by nephelometry. Interleukin (IL)-6, tumor necrosis factor (TNF)-α, ET-1, and vascular cell adhesion molecule-1 (VCAM-1) were measured using ultra-sensitive ELISA kits (brand R&D Systems, Minneapolis, MN, USA).

### 2.5. Serum Phytosterols Analyses

Markers of cholesterol absorption (campesterol and β-sitosterol) and cholesterol synthesis (lathosterol) were measured by gas chromatography/mass spectrometry using a Shimadzu GCMS-QP2010 plus version 2.5, software GCMS solution [30]. All values were corrected for total plasma cholesterol concentration.

### 2.6. RNA Isolation and Quantitative PCR

Total mRNA was extracted from mononuclear blood cells using Trizol reagent (Invitrogen Life Technologies, Carlsbad, CA, USA) according to the manufacturer’s recommendations. The mRNA content was determined spectrophotometrically and its integrity verified by visualization of 28S and 18S RNA bands in a 1% agarose gel stained with ethidium bromide. HMGCoA reductase and LDL receptor transcripts were determined by quantitative RT-qPCR and the results were normalized according to corresponding values of housekeeping β-actin. Primers were designed using primer3 Plus (http://primer3.sourceforge.net). The following sequences were used in this study: LDLr Fw:5′-GTGTCACAGCGGCGAATG-3′, Rv:5′-CGCACTCTTTGATGGGTTCA-3′; HMGCoA reductase Fw:5′-TACCATGTCAGGGGTACGTC-3, Rv:5′-CAAGCCTAGAGACATAATCATC-3 and β-actin Fw:5′-CCTGGCACCCAGCACAAT-3′, and Rv:5′-CGATCCACACGGAGTACT-3. Measurements of mRNA expression were carried out in a Rotor-Gene RG-3000 (Corbett Research, Sidney, Australia) using SuperScript™ III Platinum^®^ One-Step Quantitative RT-PCR System (Invitrogen Life Technologies, Carlsbad, CA, USA) according to the manufacturer’s instructions.

### 2.7. Statistical Analysis

Parametric tests were used for statistical analyses. Comparisons between the placebo and the PS period were analyzed by Student’s *t*-test followed by the post-hoc Wilcoxon for a nonparametric test. All results were expressed as mean ± SEM. Correlations between two variables were conducted in Pearson’s test. All analyses were performed using GraphPad Prism version 4.0 and significance level considered as *p* < 0.05.

## 3. Results

The study was initiated with 40 subjects, but two participants were excluded for presenting more than 5% body weight variation along the study. Exclusion criteria were: use of lipid-lowering medication or a prescribed diet in the last month; alcohol abuse or illicit drugs; pregnancy or breastfeeding; smoking; diabetes mellitus; thyroid, renal or hepatic diseases; or participation in another lifestyle or pharmaceutical intervention study. At screening, patients enrolled presented body mass index (25.3 ± 2.4 kg/m^2^), TC (245 ± 34 mg/dL), LDL-c concentrations (165 ± 34 mg/dL), and triglycerides levels (141 ± 53 mg/dL) as described in Table 1. In this randomized, double-blind, placebo-controlled dietary intervention trial each study period lasted 4 weeks. Initially, all participants were submitted to a 3-week run-in period in which they received the placebo product (soy milk) to test their adherence to the protocol. After the run-in period, lipid profile and body weight remained unaltered (Table 1).

After a baseline period, the individuals were randomly assigned to a placebo or phytosterol diet for 4 weeks, and subsequently, a reversed sequence was conducted. The placebo group received 400 mL of soy milk daily, whereas phytosterol group received 400 mL of soy milk enriched with 1.6 g of PS, represented as β-sitosterol-ester (78%), sitostanol-ester (13%), campesterol-ester (5.3%), and campestanol-ester (0.5%) (Table 2). Blood samples were drawn from participants in the fasting state for biochemical analysis on the last day of each period study.

All participants were advised to follow a normocaloric diet based on the NCEP-ATPIII recommendation [24]—i.e., 30% of energy as fat, <10% of energy as saturated fatty acids, and <300 mg cholesterol/day—and were recommended to avoid the consumption of products enriched with phytosterols during the study. Nutritional monitoring was conducted by a registered dietitian using a 24 h dietary recall to ensure adherence to the prescribed diet and to estimate the food intake. Soy milk was weekly supplied on the same day that body weight was measured. Patients were instructed to consume soy milk or PS-enriched soy milk twice a day, during lunch and dinner.

The body weight and BMI from subjects enrolled in the study did not change throughout the investigation (Table 3). PS-treated patients presented higher plasma concentrations of campesterol and β-sitosterol as compared to the placebo group, which indicates compliance with the diet. In PS-treated individuals, the levels of total cholesterol, LDL-c, apo-B, and triglycerides were reduced, but no change in HDL-c plasma concentrations was observed (Table 3). The reduction in plasma LDL-c levels was not associated with changes in HMGCoA-reductase or LDLR gene expression but was likely due to a decrease in dietary cholesterol absorption as shown by increased plasma levels of lathosterol (Table 3).

Since high plasma total cholesterol and LDL-c concentrations are correlated to activation of inflammatory signaling pathways and endothelial dysfunction, we also verified whether PS intake could alter the levels of inflammatory markers. As shown in Table 3, PS did not alter fibrinogen, hs-CRP, IL-6, SAA, TNFα, or VCAM-1 plasma concentrations. Nonetheless, a significant decrease in ET-1 concentration was observed after PS treatment (Table 3).

To understand whether the beneficial effects of PS would persist in different degrees of hypercholesterolemia, the patients were divided into two groups according to the median of LDL-c at baseline (≤166 mg/dL or ≥167 mg/dL). PS intake effectively reduced TC and LDL-c in both groups (Table 4). However, PS intake failed to reduce triglycerides and apo-B concentrations in those who presented LDL-c ≤ 166 mg/dL. The reductions in plasma triglycerides (−16%) and apo B (−6.5%) concentrations were observed only in patients who had higher LDL-c at baseline (≥167 mg/dL).

As plasma LDL-c concentration is an important factor involved in the regulation of ET-1 levels, we examined if the decrease in this endothelial biomarker could be influenced by the cholesterol reduction promoted by PS. Therefore, we divided the patients according to their response to PS treatment. We observed that the ET-1 responsiveness to PS was independent of the variations in LDL-c plasma concentrations. Indeed, subjects that responded to treatment and presented a reduction in LDL-c after PS intake (*n* = 18) had decreased ET-1 plasma concentration (−12%). At the same time, subjects whose LDL-c levels did not diminish upon PS treatment (*n* = 6) also presented a significant reduction (−8%) in ET-1 plasma concentration (Table 5).

**Figure 1:**
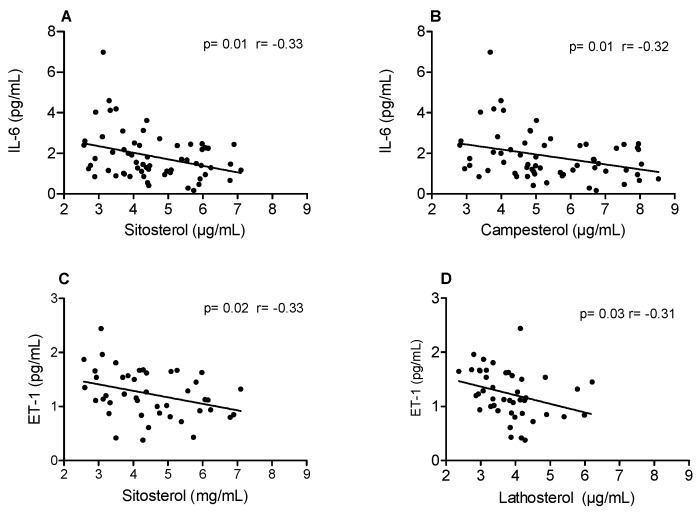
Correlation between markers of cholesterol absorption with interleukin-6 (IL-6) and markers of cholesterol absorption and synthesis with endothelin-1 (ET-1).

Confirming the beneficial impact of dietary PS, a negative correlation between plasma sterols and inflammatory markers was observed. Pearson’s correlation analyses showed a negative correlation between β-sitosterol and ET-1 plasma concentrations, as well as lathosterol, a marker of cholesterol synthesis, and ET-1 (Figure 1). Although we saw no differences in the concentrations of IL-6 between placebo and PS-treated subjects, a negative correlation was also observed between IL-6 and β-sitosterol and campesterol (Figure 1), confirming the beneficial effects of PS in counteracting inflammation.

## 4. Discussion

Our study shows that PS can reduce the risk of cardiovascular disease by mechanisms beyond its cholesterol-lowering effect. Although previous studies have shown the beneficial impact of PS on biomarkers associated with inflammation and endothelial dysfunction [31,32], the results are inconsistent possibly due to the differences in the concentration of plant sterols used in the studies and the disease stage of subjects enrolled in the investigations. Overall, our treatment reduced atherogenic lipids, but the mild cholesterol reduction observed in our study could be attributed to a smaller amount of PS given to the subjects as compared to other studies [23,32,33]. In fact, another investigation showed no reduction in plasma levels of cholesterol in subjects receiving a smaller amount of phytosterol [34]. It is also important to consider the wide variability in individual LDL-c plasma reduction in response to PS intake [14]. This variability in the response to PS treatment is mainly attributed to the individual’s sterol synthesis/absorption rate, as previously shown in type 2 diabetic patients [35]. Intriguingly, apo-B and TG plasma concentrations were reduced especially in participants presenting higher LDL-c concentrations at baseline.

As ET-1 expression is correlated with plasma levels of LDL-c [36,37,38], we tried to understand whether the decrease in ET-1 was a consequence of the reduction in LDL-c plasma concentration promoted by PS intake. Therefore, we divided the subjects into groups according to their response to PS treatment: responsive, which presented a significant decrease in plasma levels of LDL-c; and non-responsive, which displayed no apparent reduction in plasma levels of LDL-c. Interestingly, we observed that PS treatment induced a significant reduction in ET-1 plasma concentration even among non-responsive individuals. In addition, we show that the concentration of endothelin-1 has a clear inverse relationship with the increase in plasma levels of phytosterol (Figure 1), which leads us to the conclusion that phytosterols might have a direct action on the regulation of endothelin-1 in the endothelium. This result correlates with our previous study conducted in LDLr-KO mice fed a western diet showing that PS efficiently reduced atherosclerosis development, even when plasma levels of cholesterol remained elevated [23]. Therefore, the intake of PS can reduce plasma levels of ET-1 and improve endothelial function independently of its cholesterol-lowering effect.

The anti-inflammatory properties of plant sterols have been shown in studies conducted in vitro and animal models [39,40]; however, these effects are yet difficult to observe in clinical trials, especially due to the variability of the results. As an example, in healthy adults, the consumption of PS-enriched soy milk reduced lipid peroxidation and inflammatory markers [41], whereas in individuals with metabolic syndrome the PS treatment did not affect inflammatory biomarkers such as CRP, VCAM, ICAM, IL-6, CD40 ligand, E-selectin, and MCP-1 [31,32]. Likewise, in moderately hypercholesterolemic subjects, we did not find alterations in fibrinogen, hs-CRP, SAA, TNFα, VCAM-1, and IL-6 levels upon PS treatment.

Although we saw no changes in plasma levels of IL-6 between placebo and PS-treated groups, we performed a correlation analysis of this inflammatory biomarker with plasma concentrations of sitosterol and campesterol, which correlate with lower cholesterol absorption. We observed a negative correlation between the plasma levels of sitosterol or campesterol and IL-6, whereas ET-1 correlated negatively with sitosterol and lathosterol in moderately hypercholesterolemic individuals. A negative correlation between plasma levels of sitosterol and inflammatory markers was also observed in diabetic and non-diabetic subjects [42].

The consumption of PS reduces intestinal cholesterol absorption, therefore higher plasma concentrations of sitosterol and campesterol correlate with lower cholesterol absorption. As cholesterol absorption and synthesis are tightly related, we investigated if the PS treatment modulated cholesterol synthesis or LDL particle uptake by analyzing mRNA expression of HMG-CoA reductase and LDL receptor. There was no difference between the groups regarding the studied genes, showing that the increase in cholesterol synthesis, as indicated by higher plasma lathosterol concentration, and the lower LDL-c plasma concentrations might be due to post-transcriptional mechanisms. This result was also observed in patients on PS and statin treatment, which did not display changes in LDL receptor expression [33].

Importantly, this investigation was conducted in a specific population and therefore should be carefully extrapolated to the overall population, since geographic, socioeconomic, and environmental factors as well as pre-existing comorbidities must be taken into consideration. Another relevant point to be considered is that the daily consumption assessment by itself can offer its own limitations, as self-reported 24 h dietary questionnaires usually add bias such as under/over-reporting of information. On the other hand, this research shows a beneficial effect of PS beyond its cholesterol-lowering effect, which reaffirms the importance of adherence to the main Guidelines established by Health Organizations, such as the European Society of Cardiology (ESC) [43], as part of the treatment of moderated hypercholesterolemia.

## 5. Conclusions

In conclusion, we showed that the consumption of plant sterols reduced plasma levels of ET-1 independently of the cholesterol-lowering effects of plant sterols. These results contribute to the comprehension of the effect of plant sterols on endothelial function and prevention of cardiovascular diseases.

## Figures and Tables

**Table 1:** Subjects characteristics at baseline.

Parameter	Screening	After Run-in Period
Age (years)	58 ± 12	58 ± 12
Weight (Kg)	64 ± 10	64 ± 9
BMI (kg/m^2^)	25.3 ± 2.4	25.4 ± 2.3
Total cholesterol (mg/dL)	245 ± 34	249 ± 37
Triglycerides (mg/dL)	141 ± 53	142 ± 10
LDL-c (mg/dL)	165 ± 34	173 ± 31
HDL-c (mg/dL)	49 ± 12	47 ± 10

BMI, body mass index; LDL, low-density lipoprotein. HDL, high density lipoprotein; *n* = 38. Data shown as mean ± SD.

**Table 2:** Soy milk nutritional composition per portion (200 mL).

Nutritional Composition	Soy Milk	Soy Milk + PS
Energy (kcal)	138	144
Protein (g)	6.5	6.5
Total fat (g)	4.4	5.0
Polyunsaturated fat	2.3	2.5
Monounsaturated fat	1.0	1.1
Saturated fat	0.7	0.9
Trans fatty acid	0	0
Cholesterol (mg)	0	0
Carbohydrates (g)	18.2	18.2
Total sugar	14.1	14.1
Lactose	0	0
Phytosterols (g)	0	0.8
β-sitosterol-ester		0.63
Sitostanol-ester		0.10
Campesterol-ester		0.05
Campestanol-ester		0.005
Sodium (g)	0.1	0.1

**Table 3:** Body weight, BMI, biochemical analysis, mRNA, and plasma sterol concentrations of moderately hypercholesterolemic subjects after supplementation.

	*n*	Placebo	Phyto	*p*
Weight (kg)	38	64.9 ± 10.2	65.1 ± 10.3	0.08
BMI (kg/m^2^)	38	25.4 ± 0.4	25.4 ± 0.4	0.89
Total cholesterol (mg/dL)	38	261 ± 7.1	244 ± 5.8 *	<0.001
HDL-c (mg/dL)	38	46 ± 1.7	48 ± 1.9	0.54
LDL-c (mg/dL)	38	183 ± 5.9	169 ± 5.2 *	0.001
Apo B (mg/dL)	38	126 ± 3.7	118 ± 3.2 *	0.006
Triglycerides (mg/dL)	38	154 ± 10	133 ± 7 *	0.008
Fibrinogen (mg/dL)	25	3.6 ± 0.5	3.5 ± 0.5	0.79
hs-CRP (mg/L)	30	3.1 ± 0.4	3.1 ± 0.4	0.96
SAA (mg/L)	33	6.78 ± 0.52	5.94 ± 0.51	0.16
IL-6 (pg/mL)	33	2.69 ± 1.03	2.24 ± 1.01	0.25
TNFα (pg/mL)	36	1.35 ± 0.07	1.28 ± 0.06	0.19
VCAM-1(ng/mL)	38	469 ± 127	472 ± 118	0.69
ET-1 (pg/mL)	24	1.31 ± 0.09	1.13 ± 0.09 *	0.02
HMGCoAr/Actb (fold change)	21	1.10 ± 0.20	1.05 ± 0.16	0.70
LDLr/Actb (fold change)	17	1.24 ± 0.23	1.26 ± 0.21	0.66
Plasma Sterols Expressed as µg/ mg Cholesterol				
Lathosterol	38	1.53 ± 0.09	1.69 ± 0.06 *	0.01
Campesterol	38	1.96 ± 0.12	2.34 ± 0.11 *	0.02
β-sitosterol	38	1.64 ± 0.09	2.02 ± 0.09 *	<0.001
Lathosterol/campesterol ratio	38	0.85 ± 0.05	0.76 ± 0.03 *	<0.001
Lathosterol/β-sitosterol ratio	38	1.03 ± 0.05	0.88 ± 0.04 *	<0.001

hs-CRP, high-sensitivity C-reactive protein; SAA, serum amyloid A; IL-6, interleukin-6; TNFα, tumor necrosis factor-α; ET-1, endothelin-1; VCAM-1, vascular cell adhesion molecule-1. Data shown as mean ± SEM. Placebo vs. Phyto * *p* < 0.05.

**Table 4:** Plasma lipids and apo B concentration according to the mean of LDL-c plasma concentration at baseline, of moderately hypercholesterolemic patients.

	LDL-c ≤ 166 mg/dL ^1^			LDL-c > 167 mg/dL ^2^		
	Placebo	Phyto	% Change	Placebo	Phyto	% Change
Triglycerides (mg/dL)	146 ± 58	134 ± 45	−1.9	164 ± 61	132 ± 41 *	−16.0
Total cholesterol (mg/dL)	243 ± 34	230 ± 26 *	−4.7	283 ± 44	263 ± 37 *	−6.4
LDL-c (mg/dL)	166 ± 24	154 ± 20 *	−6.2	202 ± 39	186 ± 33 *	v6.8
Apo-B (mg/dL)	115 ± 12	109 ± 13	−4.3	140 ± 25	129 ± 20 *	−6.5

^1^*n* = 21, ^2^ *n* = 17; Data shown in mean ± SD. Placebo × Phyto * *p* < 0.05.

**Table 5:** Endothelin-1 plasma concentration of subjects according to LDL-c response to phytosterol intake.

Response to Treatment	Placebo	Phytosterol	% Change
PS Responsive (*n* = 18)	1.41 ± 0.38	1.21 ± 0.44 *	−12
PS Non-Responsive (*n* = 6)	1.00 ± 0.39	0.91 ± 0.35 *	−8

Data shown in mean ± SD; * Placebo × Phyto: *p* < 0.05; Student *t* test.
